# Transfer Length vs. Slip of Prestressed Fiber-Reinforced Polymer Reinforcement

**DOI:** 10.3390/polym15051190

**Published:** 2023-02-27

**Authors:** Aidas Jokūbaitis, Juozas Valivonis

**Affiliations:** Department of Reinforced Concrete Structures and Geotechnics, Faculty of Civil Engineering, Vilnius Gediminas Technical University, Sauletekio Av. 11, LT-10223 Vilnius, Lithuania

**Keywords:** transfer length, slip, fiber-reinforced polymer, prestress, bond

## Abstract

A comprehensive analysis of the relationship between transfer length and slip of different types of prestressed fiber reinforced polymer (FRP) reinforcement is provided. The results of the transfer length and slip together with the main influencing parameters of approximately 170 specimens prestressed with different FRP reinforcement were collected. After the analysis of a larger database of transfer length versus slip, new bond shape factors were proposed for carbon fiber composite cable (CFCC) strands (α = 3.5) and carbon fiber reinforced polymer (CFRP) bars (α = 2.5). It was also determined that the type of prestressed reinforcement has an influence on the transfer length of the aramid fiber reinforced polymer (AFRP) bars. Therefore, α = 4.0 and α = 2.1 were proposed for AFRP Arapree bars and AFRP FiBRA and Technora bars, respectively. Moreover, the main theoretical models are discussed together with the comparison of theoretical and experimental transfer length results based on the slip of reinforcement. Additionally, the analysis of the relationship between transfer length and slip and the proposed new values of the bond shape factor α have the potential to be introduced in the production and quality control processes of precast prestressed concrete members and to stimulate additional research that increases the understanding of the transfer length of FRP reinforcement.

## 1. Introduction

The corrosion of prestressed steel is one of the main concerns that affect the durability of prestressed concrete structures exposed to corrosive environments, such as offshore platforms, marine structures, bridges, parking garages, and railway sleepers [1,2]. The loss of steel to corrosion is accelerated in chloride-rich environments. Ingress of humidity and aggressive substances into structures can appear through imperfections in the concrete structure, cracks, or at the ends of prestressed concrete members, where the pretensioned steel reinforcement is usually uncovered [1]. The expansive nature of steel corrosion can damage the concrete surrounding reinforcement. Therefore, the bond between reinforcement and concrete will also be damaged. Additionally, the reinforcement cross section is reduced over time as a result of the corrosion process and is unable to take the required tension. Therefore, the initial design assumptions may not be satisfied, leading to excessive deformation of the prestressed concrete member, causing a reduction in durability which can lead to premature failure.

A solution to the corrosion problem within concrete structures is replacing the steel reinforcement with noncorrosive FRP reinforcement (CFRP and AFRP). The high strength of FRP reinforcement allows it to be applied in prestressed concrete structures. Furthermore, a low modulus of elasticity reduces prestress losses due to the relaxation of prestressed reinforcement and creep and shrinkage of concrete. Therefore, FRP reinforcement becomes an attractive material to be used in prestressed concrete structures.

The bond between reinforcement and concrete is one of the main parameters that describe the behavior of concrete members [3,4]. The bond of the FRP reinforcement depends on the type and mechanical properties of the FRP reinforcement, the surface properties [5,6], and the concrete properties [7]. These main parameters are also applicable to the prestressed concrete members where the prestressing force in the pretensioned reinforcement is transferred to the concrete through the bond. It depends on adhesion (chemical bond), the Hoyer effect (wedge action), and mechanical interlocking. The bond due to adhesion is very small if FRP reinforcement is used [8]. On the contrary, the Hoyer effect influenced by the large Poisson ratio and the high axial strain capacity of the FRP material together with the mechanical interlock influenced by the roughness of the reinforcement surface are the main parameters describing the bond between prestressed FRP reinforcement and concrete [8]. According to Nanni et al. [9], the influence of the surface properties of the FRP reinforcement on the bond may be small if the Poisson ratio is large. However, mechanical interlock and friction depend on the surface roughness of the reinforcement. If the bond between reinforcement and concrete is not ensured or damaged, the prestress loses its purpose to increase the stiffness of the concrete member, which can also lead to failure. Therefore, it is essential to properly assess the anchorage zone of the prestressed reinforcement. The main parameter describing the anchorage zone of the prestressed concrete member during pretensioned reinforcement release is called the transfer length (L_t_). The transfer length is defined as the length from the free end of the member to the point along the length of the member where the effective prestress in reinforcement is fully transferred to the concrete during reinforcement release. The transfer length is mainly dependent on the properties of the prestressing reinforcement and concrete. A detailed review of the parameters that influence the transfer length of different FRP reinforcements is presented in [10].

Some authors performed research comparing the transfer length of pretensioned seven-wire steel strands and different FRP reinforcements. The average transfer length of most FRP reinforcements tested was lower compared to steel reinforcement. Ehsani et al. [11] determined that the transfer lengths of the AFRP bars were 60–94% of the average transfer length of steel strands. Furthermore, the transfer lengths of the CFRP Leadline bars were about 80% of the steel transfer length. In [12], it was estimated that the transfer length of CFRP bars and CFCC strands was approximately 25% higher and 50% lower compared to steel strands, respectively. Additionally, the results of finite element analysis taking into account the surface conditions of sanded BFRP bars [13] and AFRP bars and CFCC strands [14] also showed a lower transfer length compared to steel strands. Other researchers concluded that the lower transfer length of the FRP reinforcement compared to the steel strands is influenced by the properties of the FRP reinforcement. In particular, higher friction is induced between the reinforcement and concrete during prestress transfer due to the lower modulus of elasticity of FRP reinforcement compared to the steel strands. The friction appears when the prestressed reinforcement is released and shear stresses are induced by the Hoyer effect and mechanical interlock together with longitudinal stresses [15]. The shorter transfer length of the FRP reinforcement compared to steel strands is one of the advantages for practical applications. However, the stress concentration at the end of the member is significantly higher compared to the steel strands. Therefore, additional solutions should be applied to the anchorage zone of the prestressed FRP reinforcement to decrease the probability of concrete cracking by increasing the confinement of the concrete. The splitting of concrete during the release of prestressed reinforcement can be controlled with a sufficient concrete cover. According to [10], the minimum concrete cover for CFRP, AFRP, and GFRP reinforcement is 1.9·Ø and 2.8·Ø, respectively, and the sufficient concrete cover for the CFCC strand is 4·Ø. Additionally, the confinement of concrete can be increased by introducing shear reinforcement in the anchorage zone of prestressed FRP reinforcement. Therefore, the introduction of shear reinforcement governs the reduction of the transfer length of prestressed FRP reinforcement [12,15].

In recent decades, many experimental investigations have been performed investigating the transfer length of different prestressed FRP reinforcements. However, there are different experimental methods for determining transfer lengths: direct and indirect. The detailed analysis of the database results in the transfer length of different FRP reinforcements measured with the direct methods is presented in [10,16]. The indirect method of determining the transfer length by measuring the slip of the FRP reinforcement during pretensioned reinforcement release was investigated by several authors: for the CFCC strands [12,15,17], for the CFRP bars [12,15,17,18,19,20], for the AFRP bars [21,22], and for the BFRP bars [23]. However, the manufacturing technology allows for the production of FRP reinforcement with different strengths, surface properties, shapes, and modulus of elasticity. Therefore, there is still a need for a deeper understanding of the transfer length of different FRP reinforcements. Therefore, this article presents a comparative analysis of the collected database of transfer length and slip of different types of pretensioned FRP reinforcement.

## 2. Methods of Measuring Transfer Length

There are several methods to determine the transfer length of the prestressed reinforcement. Therefore, this section describes different methods for measuring and determining transfer lengths for prestressed reinforcement.

Transfer length can be measured by applying electrical resistance strain gauges (ERSG) to the reinforcement surface along the reinforcement. The strain gauges are attached to the reinforcement surface with a selected distance [8]. Strains in the reinforcement can be monitored throughout the whole process of manufacturing the specimen, and therefore, the loss of prestress can be determined. For the determination of the transfer length, the readings of strain gauges are taken immediately before and after the release of the prestressed reinforcement. Transfer length can be determined from the relationship between the strains in the reinforcement and the length of the specimen. However, this type of strain measurement has some disadvantages. It is difficult to maintain the strain gauges intact during the manufacturing process due to the pouring and vibration of the concrete. Therefore, some strain gauges can be damaged and part of the results may be lost. Furthermore, strain gauges must be checked if they are constantly working throughout different technological processes of specimen production. Strain gauges are usually protected with additional cover to avoid possible damage as much as possible. However, it interrupts the bond between reinforcement and concrete, and, therefore, the transfer length may be longer and it can be determined incorrectly. Therefore, it can have a negative impact on the overall design of the prestressed concrete member.

Other methods based on the use of DEMEC (demountable mechanical strain gauge) points glued to the concrete surface at the level of the prestressed reinforcement are used to measure the strain of the concrete surface using a DEMEC gauge [24,25]. This method does not affect the bond between concrete and reinforcement. The DEMEC points are stainless steel circular discs with a 1 mm pinhole in the center to provide precise measurements. A DEMEC gauge with an accuracy of 0.001 mm is usually used to measure the distance between points immediately before and after the prestressed reinforcement release. Then the concrete surface strain can be determined according to the difference in the DEMEC gauge results. The transfer length of the prestressed reinforcement is determined from the relationship between the concrete strain and the length of the specimen, as was presented in the case of using strain gauges on the surface of reinforcement. Despite the main advantage of this method that it does not disrupt the bond between the reinforcement and the concrete, it also has some disadvantages. The measurement of the distance between DEMEC points is sensitive to the position of the DEMEC gauge (inclination, vertical, and horizontal positioning). Additionally, it is very dependent on the person who performs the measurement, and it involves the possibility of human error. These disadvantages can be solved by using ERSGs glued to the concrete surface instead of using DEMEC points. The strain measurements on the concrete surface show the lag effect of stresses dispersed throughout the concrete between the reinforcement and the surface of the specimen. Therefore, the concrete cover influences the strain. Additionally, the results of long-term concrete surface strain measurements are affected by the creep and shrinkage of concrete.

A similar measurement of concrete strain on the surface of the prestressed concrete specimen using the Whittemore gauge point system can be used. This method requires the attachment of gauge points to the surface of the specimen. As an alternative to gluing the DEMEC points to the specimen by hand, contact point inserts can be screwed onto the inside surface of the formwork before placing the concrete. The screws are removed before the formwork is removed so that the inserts remain in place. The measurement of concrete strain is performed the same as in the case of DEMEC points [11].

The optical speckle technique was used by [26,27,28] to measure the transfer length of the prestressed reinforcement. Speckle is generated by illuminating a rough surface with coherent light. The randomly reflected waves interfere with each other, resulting in a grainy image. The speckle pattern moves when the increase of stress induces the deformation of the member. Then the movement of the speckle pattern is determined by converting the deformation on the surface to the strain difference. The surface strain is determined by comparing the displacement of the grainy speckle pattern image taken before and after the prestress transfer. The application of the speckle pattern technique requires minimal surface preparation, it is compatible with almost all types of rough surfaces, and it has high resolution. Additionally, it solves the human error issue in the case of transfer length measurement with DEMEC and Whittemore gauges. However, the position of the laser speckle device between the initial reading and the final reading must be maintained very precisely. Therefore, the results are very sensitive to small changes in the measurement position. Additionally, the accuracy of the measurement is very sensitive to changes in the concrete surface. According to [26], it seems that the painted concrete surface may solve this problem.

Transfer length determination can be performed by measuring the reinforcement strain using fiber optic sensors. The methods used for the production of FRP reinforcement allow the installation of very thin fiber optic sensors in the center of the FRP reinforcement along the longitudinal axis [29,30]. Therefore, the diameter of the reinforcement is almost unchanged, and there is no distortion of the bond between the reinforcement and the concrete, as is the case with strain gauges glued to the reinforcement surface.

Jeon et al. [30] compared three different methods to determine the transfer length of the prestressed concrete members. Strains on the concrete surface at the level of prestressed reinforcement and on the surface of the reinforcement were measured with glued ERSG. Additionally, Smart Strand was used for measuring the strain along the reinforcement. The Smart Strand is a regular seven-wire steel strand with CFRP core wire with an embedded fiber optic sensor. A fiber optic sensor with a certain number of fiber Bragg gratings (FBG) is installed during the production of the CFRP bar. Jeon et al. [30] concluded that the Smart Strand technique for determining the transfer length is the most reliable. The difference between the transfer length measured with Smart Strands and ERSGs on the concrete surface was only 3%, and both methods gave sufficiently reliable results. However, the use of ERSGs on the reinforcement surface gave unreliable transfer length results due to deterioration and reading errors.

The relationship between reinforcement strain and the length of the specimen shows increasing strains starting from the end of the specimen with a constant plateau deeper into the specimen. The point where the strain increase stops, and becomes constant is the point of transfer length. However, this zone has nonlinear behavior and it becomes harder to determine the exact transfer length. Three types of strain evaluation through the length of the member for determining the transfer length are proposed in the literature. In the analysis of measured strains, several researchers used the length between the end of the member and the intersection point of the variable strain region and the constant strain plateau as the transfer length [31]. This method is called the “100% constant strain method”. Other researchers determined the transfer length by the location at which the varying strain region intersects a horizontal line corresponding to 95% of the constant strain plateau [32,33]. This method is called the “95 percent constant strain method”. In the “slope-intercept” method, the transfer length is the distance from the end of the member to the point of intersection of the straight line fitting the measured strains within the transfer zone and the constant strain plateau. The slope intercept method has the disadvantage that a judgement must be made about how many strain readings should be included in the regression analysis process for each line. Up until now, the most widely used approach for transfer length determination has been the “95 percent constant strain method”.

There is a strong correlation between the transfer length and the slip of the prestressed reinforcement during transfer. The slip is the cumulative effect of the small movement of prestressed reinforcement relative to the concrete during release. If the applied stress is known, measuring the slip of the prestressed reinforcement relative to the concrete surface enables estimating the length of the prestressed strands that contribute to deformation [20]. Therefore, the slip of the prestressed reinforcement at the end of the concrete specimen can be used to indirectly predict the transfer length. Additionally, measurement of the slip of prestressed reinforcement during transfer can be used as an approximate method to verify the transfer length. Such measurements are much faster and simpler to perform, making the recording of these a viable approach to quality assurance of the transfer length. However, when relating the slip of prestressed reinforcement with the transfer length, several assumptions are made: the stress varies linearly from zero at the beam end to a maximum value within a distance equal to the transfer length and the plain sections remain plain. In addition, the variation of the slip results is usually greater compared to other measurement methods.

Krem [19] determined the transfer length of CFRP bars by measuring the concrete strain profile and the slip of the prestressed bars during release. Both methods were determined to show a similar tendency in the experimental results. However, the transfer length determined by measuring the concrete strain profile was slightly higher compared to the slip results. It was related to the development of the assumption that the reinforcement stress varies linearly from zero at the end of the beam to the full effective prestressing stress at the end of the transfer length. The results of the concrete strain profile proved the opposite. The stress increase reduces near the end of the transfer length, and the stress distribution becomes nonlinear. Furthermore, the transfer length determined according to the concrete strain profile method depends on the experimental measurements. The transfer length determined according to the slip method depends on the mechanical properties of the prestressing reinforcement and the measured displacement.

The slip method to determine the transfer length of the prestressed reinforcement is the easiest and fastest method compared to other methods. It requires the linear variable differential transducer (LVDT) gauge to measure the slip of reinforcement during the transfer of prestress force into the concrete. Additionally, parallel measurement of the prestress force during release is required. Furthermore, the gradual release of prestressed reinforcement allows stepwise measurements allowing the determination of a bond law that is directly related to the transfer length [34]. In addition, this method gives transfer length results with sufficient accuracy, making it very attractive for manufacturers of precast and prestressed concrete structures. Therefore, the transfer length results determined according to the slip of different prestressed FRP reinforcements are presented in this article.

## 3. Theoretical Models

**Figure 1:**
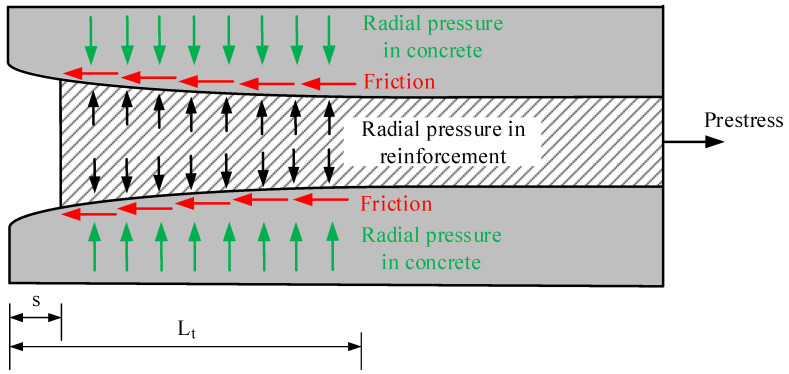
Mechanism of pretensioned reinforcement release.

The prestressed concrete members overcome many stages during their design working life. One of the first stages is the production of the member. At first, the reinforcement is tensioned and the diameter of the bar decreases. Then the concrete is poured, cured, and the bond is formed between the reinforcement and concrete. Before the release of the prestressed reinforcement, chemical adhesion plays a role in this bond. Reinforcement shortens during the release of reinforcement from the abutments. The contraction of reinforcement destroys the adhesion, and the bond between reinforcement and concrete is ensured by mechanical interlock, which depends on the surface conditions of the reinforcement, and friction, which is caused by the Poisson effect and the surface conditions of the reinforcement. When reinforcement is released, a slip occurs at the end of the member due to a loss of stress within the transfer length. Therefore, at the end of the reinforcement, the stresses become zero. Additionally, the pretensioned reinforcement tries to regain its initial diameter during release. The concrete around the reinforcement restricts the expansion of the prestressed reinforcement, and the Hoyer or wedge effect [35] appears at the end of the member. Therefore, a radial pressure is induced in the concrete perpendicular to the surface between the reinforcement and concrete. The pressure created as the reinforcement attempts to expand produces the normal forces needed to create a friction reaction during the slip of the reinforcement (Figure 1). However, the influence of the Hoyer effect for pretensioned FRP reinforcement is slightly different compared to steel strands. This is influenced by the fact that FRP reinforcement is an anisotropic material that has good mechanical properties along the fibers and low transverse strength and stiffness (perpendicular to the fibers). Furthermore, the stiffness of the FRP reinforcement is lower compared to steel. Therefore, the Hoyer effect and the resulting frictional stresses are directly related to the Poisson ratio and the transverse modulus of elasticity of the FRP bar and concrete, and to the friction coefficient at the reinforcement–concrete interface. Therefore, the low transverse modulus of elasticity can increase the friction component of the bond between the FRP reinforcement and concrete during the release of the prestressed reinforcement. Furthermore, additional surface roughness in the form of sand coating, indentations, fiber roving, etc., increases the friction and mechanical interlocking components of the bond of FRP reinforcement. The influence of the initial prestress, concrete strength at transfer, type of prestress transfer (sudden or gradual), type, diameter, and surface properties of reinforcement, consolidation, and protective cover of concrete also affects the transfer length. Therefore, according to [11,12,13,14], the transfer length of the FRP reinforcement can be lower compared to steel strands.

Friction between concrete and the reinforcement depends on the coefficient of friction, the properties of the reinforcement surface, and the strength of the concrete.

The reinforcement displacement appears only within the transfer length during release. However, there is no displacement of the reinforcement in the middle of the prestressed concrete member. Therefore, the compatibility of the strains between the prestressed reinforcement and the concrete (ε_c_ = ε_s_) can be considered.

The measurement of the slip of the reinforcement is an indirect method to determine the transfer length in prestressed concrete members. Many formulas are proposed that describe the relationship between the slip(s) of pretensioned steel strands during release and the transfer length (L_t_) (Table 1). However, no equation was proposed for anisotropic FRP reinforcement.

Guyon [36] proposed a theoretically derived relationship (Equation (1)) between the transfer length (L_t_) and the slip(s) of the pretensioned steel reinforcement taking into account the modulus of elasticity of the prestressing reinforcement (E_p_, the assumed isotropic steel), the reinforcement stress immediately before release (f_pi_), and the bond shape factor (α).

A similar equation (Equation (2)) is presented in [37], only the bond shape factor is taken as α = 2.08. However, this relationship is applied to precast concrete products prestressed with steel wires or strands.

Rose and Russell [38] proposed a relationship between transfer length and reinforcement slip (Equation (3)) based on Equation (1) and experimental results of the transfer length of 12.7 mm diameter seven-wire steel strands. Equation (1) with bond shape factor α = 2 was modified by introducing the sum of the mean difference (17.78 mm) and a standard deviation (119.38 mm) of strand slip.

Balazs [39,40] proposed two relationships between the transfer length and slip of a 12.7 mm diameter seven-wire steel strand (Equations (4) and (5)). Both equations are theoretically derived with empirical coefficients determined according to the experimental results of the bond stress–slip relationship. Equation (4) takes into account the concrete compressive strength at transfer (f_ci_) and reinforcement diameter (Ø). Equation (5) was derived from taking into account the concrete compressive strength at transfer (f_ci_), the reinforcement stress immediately before release (f_pi_), and the modulus of elasticity of the prestressing reinforcement (E_p_). However, these relationships (Equations (4) and (5)) were derived only for one type and diameter of steel strand. Therefore, with the change in diameter and type of reinforcement, new empirical coefficients that describe the bond should be determined from the experimental bond stress–slip curves.

Marshall and Krishnamurthy [41] proposed Equation (6) which only evaluates the slip of reinforcement during the release of the prestress and the empirical coefficient proposed for a 12.7 mm seven-wire steel strand. It is evident that the transfer length and slip of the prestressed reinforcement depend on more influencing parameters. Therefore, the simplicity of Equation (6) suggests that it is difficult to apply it to different types of prestressed reinforcement with different diameters and surface conditions.

**Table 1:** Summary of the relationships between transfer length and slip of steel strands.

Reference	Equation	Equation No.	Notes
[36]	$L_{t}=\frac{\alpha \cdot s\cdot E_{p}}{f_{pi}}$	(1)	α—bond shape factor s—end slip E_p_—modulus of elasticity of the prestressing reinforcement f_pi_—reinforcement stress immediately before release
[37]	$L_{t}=\frac{s\cdot E_{p}}{0.48\cdot f_{pi}}$	(2)	s—end slip f_pi_—reinforcement stress immediately before release E_p_—modulus of elasticity of the prestressing reinforcement
[38]	$L_{t}=\frac{2\cdot s\cdot E_{p}}{f_{pi}}+137.16$	(3)	s—end slip f_pi_—reinforcement stress immediately before release E_p_—modulus of elasticity of the prestressing reinforcement
[40]	$L_{t}=105\cdot \emptyset \cdot \sqrt[{4}]{\frac{s^{3/2}}{f_{ci}}}$	(4)	s—end slip Ø—reinforcement diameter f_ci_—concrete compressive strength at transfer
[39]	$L_{t}=\frac{111\cdot s^{0.625}}{f_{ci}^{0.15}\cdot {\left(\frac{f_{pi}}{E_{p}}\right)}^{0.4}}$	(5)	s—end slip f_ci_—concrete compressive strength at transfer f_pi_—reinforcement stress immediately before release E_p_—modulus of elasticity of the prestressing reinforcement
[41]	$L_{t}=\sqrt{\frac{s}{K}}$	(6)	s—end slip K = 0.0000035 mm−1 for 12.7 mm 7-wire strand

**Figure 2:**
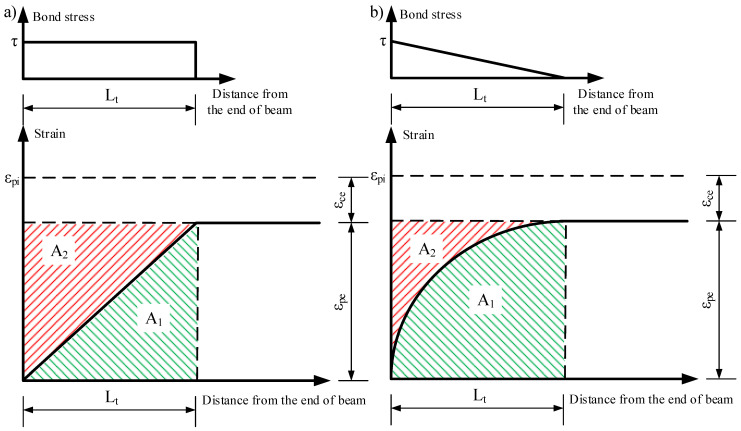
Strain variation over transfer length (**a**) linear and (**b**) parabolic, and corresponding bond stress distribution.

The coefficient α in Equation (1) proposed by Guyon [36] represents the shape factor of the bond stress distribution along the transfer length. Guyon [36] considered that, in the case of a constant bond stress distribution and the linear distribution of the prestressing and concrete strains, the bond shape factor α = 2 (Figure 2a); and in the case of a linear bond stress distribution and the parabolic distribution of the prestressing and concrete strains, the bond shape factor α = 3 (Figure 2b). Additionally, the bond shape factor is the ratio between the sum of the bond areas A_1_ and A_2_ and the area of A_2_ (Figure 2). The bond shape factor depends on the mechanical properties and quality of concrete, the type and surface properties of the reinforcement, or the properties of the reinforcement that influence the bond between the reinforcement and concrete, thus generating different strain distributions. The FRP reinforcement being an anisotropic material has a lower stiffness in the transverse direction compared to the longitudinal direction. Additionally, the manufacturing technology of FRP reinforcement allows different types of surface properties (sanded, spirally wounded, spirally indented, braided, helical plain) that increase the bond between reinforcement and concrete. Therefore, these distinctive properties of FRP reinforcement (compared to steel strands) increase friction and mechanical interlocking during the release of prestressed reinforcement and can reduce the transfer length.

There are many proposals for the bond shape factor (α) for steel strands in the literature. Therefore, the coefficient α describing the bond stress distribution along the transfer length of the steel strands varies between 1.5 and 4 [42]. However, different FRP reinforcements lack information about the bond shape factor. Table 2 provides the values of α found in the literature for different FRP reinforcements.

Crosset et al. [43] determined that the coefficient α is between 2.82 and 3.32 for BFRP bars with a sand-coated surface. However, these values were obtained only from two tested specimens. The coefficients were determined at the live end of the beams. Additionally, it was determined that the bond shape factors for both beams did not vary significantly with time.

Mahmoud [12] investigated the bond characteristics of CFRP Leadline bars and CFCC strands. Using the initial prestress strain (ε_pi_) and the measured transfer length (L_t_) for each specimen, he determined the average value of α = 2.91 and α = 2.48 for CFRP Leadline bars and CFCC strands, respectively.

Taerwe and Pallemans [21] investigated the transfer length of sand-coated AFRP bars. According to the experimental results of the relationship between transfer length and the slip of reinforcement, it was determined that the coefficient α ranges between 2.16 and 4.80 with a mean value of 3.51 for the AFRP bars with a nominal diameter of 7.5 mm and 3.02 for the AFRP bars with a nominal diameter of 5.3 mm. However, according to [21], when the results of the prism 7.5S/N/50/3 are considered unacceptable and when the results of the high strength concrete (HSC) are discarded, the significant difference between both groups disappears, resulting in an overall mean value of α = 3.03.

## 4. Results

### 4.1. Results of Database of Transfer Length versus Slip

A review of the literature on the experimental results of the relationship between transfer length and the slip of different types of pretensioned FRP reinforcement was performed. The data from approximately 170 specimens were collected in the database provided in Appendix A (Table A1, Table A2, Table A3 and Table A4 in Appendix A). In particular, 27 of 46, 27 of 60, 53 of 60, and 4 of 4 specimens prestressed with CFCC (Table A1), CFRP (Table A2), AFRP (Table A3), and BFRP (Table A4) reinforcement were found with the transfer length and corresponding slip results, respectively. The data provided in Appendix A (Table A1, Table A2, Table A3 and Table A4) consist of the original marking of the specimen, type, and surface properties of FRP reinforcement; specimen type and dimensions (b × h × l—width and height of the cross-section and length of the specimen); type of prestressed reinforcement release; presence of shear reinforcement; protective concrete cover (c); reinforcement diameter (Ø); the cross-sectional area of one prestressed bar (A_p_); tensile strength (f_pu_); modulus of elasticity (E_p_); initial stresses (f_pi_) of reinforcement; the ratio between initial stresses and tensile strength of reinforcement (f_pi_/f_pu_); concrete compressive strength at transfer (f_ci_); average transfer length (L_t_); and slip(s) of pretensioned reinforcement. The summary of material mechanical and geometric properties of the database of transfer length and slip of different types of FRP reinforcement is provided in Table 3.

### 4.2. Analysis of Experimental Results

**Figure 3:**
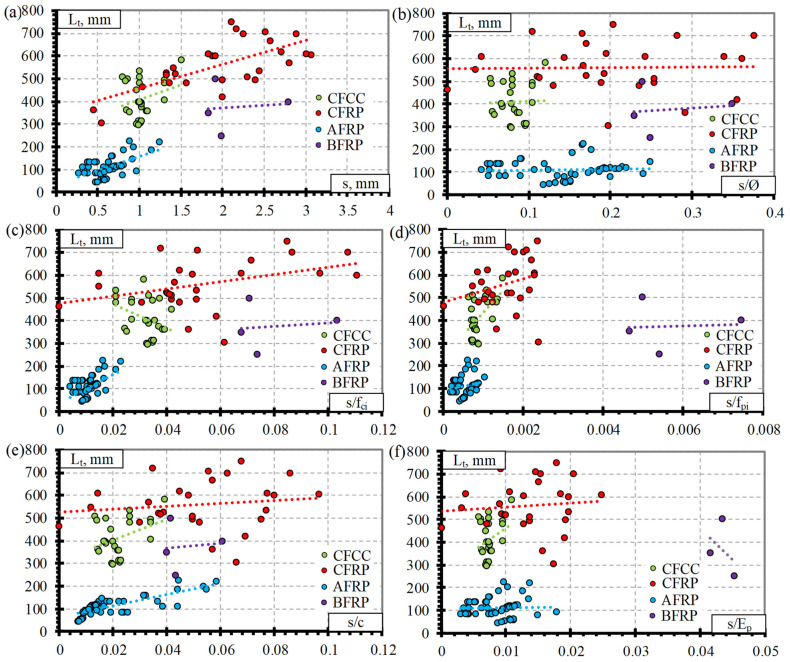
Comparison between transfer length of different FRP reinforcements and: (**a**) slip, (**b**) s/Ø, (**c**) s/f_ci_, (**d**) s/f_pi_, (**e**) s/c, (**f**) s/E_p_.

This section presents the experimental results of the transfer length and slip of different types of pretensioned FRP reinforcement (CFCC, CFRP, AFRP, and BFRP). Figure 3 shows the influence of slip(s) (Figure 3a), the ratio between slip and reinforcement diameter (s/Ø) (Figure 3b), slip and concrete compressive strength at transfer (s/f_ci_) (Figure 3c), slip and initial stress in pretensioned reinforcement (s/f_pi_) (Figure 3d), slip and concrete protective cover (s/c) (Figure 3e), and slip and modulus of elasticity of FRP reinforcement (s/E_p_) (Figure 3f). The summary of the main influential parameters (Ø, f_ci_, f_pi_, c, and E_p_) in Figure 3 are presented in Table 3. The slip of the pretensioned reinforcement depends on these influential parameters, and therefore, the comparison of the experimental results is presented as the ratio between the slip and each of the influential parameters.

It can be seen that there is a direct relation between the transfer length and the slip of different types of pretensioned FRP reinforcement and, therefore, with increasing slip, the transfer length also increases (Figure 3a). The higher the slip of reinforcement at the end of the member during the transfer of prestress, the higher the bond damage at the end of the member. Therefore, the complete prestress transfer moves further into the member, and the transfer length becomes longer.

The comparison of transfer length and s/Ø (Figure 3b) of different FRP reinforcements shows that the influence of diameter is small. However, it is also evident that the scatter of the results is high. As can be seen from Figure 3d, the transfer length increases with the increase in s/f_pi_ for CFCC, CFRP, and AFRP reinforcement. Similar trends can be observed with increasing s/c (Figure 3e) and s/E_p_ (Figure 3f). Furthermore, with an increase of s/f_ci_, the transfer length of CFRP and AFRP bars increases, and the transfer length of CFRP bars decreases (Figure 3c). Therefore, it is clear that the transfer length is not dependent only on one influential parameter. It should also be mentioned that the scatter of the results of CFCC strands and CFRP bars is higher (Figure 3c).

The influence of different parameters on the transfer length versus slip for the BFRP bars is low, and there is no clear trend in the results (Figure 3). It is related to the low number of specimens and the experimental results of the transfer length and slip of the BFRP bars. Therefore, in the literature, no or little range of influential parameters (Table 3) has been found.

Regarding the relationship between transfer length and the slip of reinforcement, it should be clear that one or several influential parameters (Ø, f_ci_, f_pi_, c, E_p_) will affect the slip of prestressed reinforcement at transfer and, consequently, the transfer length. Therefore, the analysis of the relationships provided in Figure 3b–f is not always straight forward.

### 4.3. Derivation of Theoretical Coefficients

**Figure 4:**
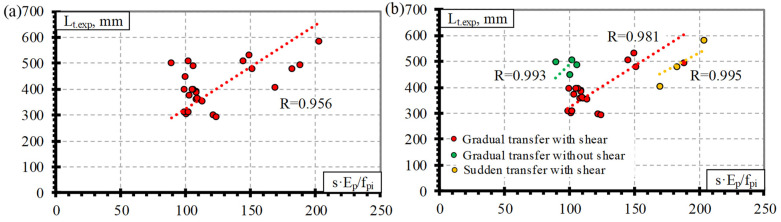
Comparison between the transfer length of the CFCC strands and s·E_p_/f_pi_: (**a**) all results, (**b**) effect of reinforcement release type and shear reinforcement.

**Figure 5:**
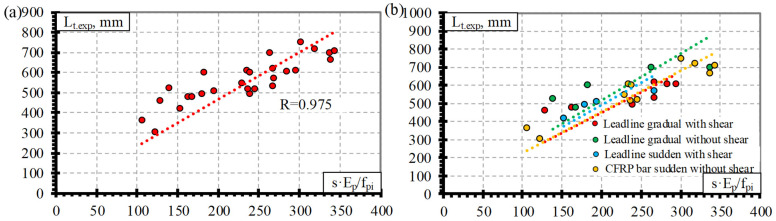
Comparison between the transfer length of CFRP bars and s·E_p_/f_pi_: (**a**) all results, (**b**) effect of reinforcement type, reinforcement release type and shear reinforcement.

**Figure 6:**
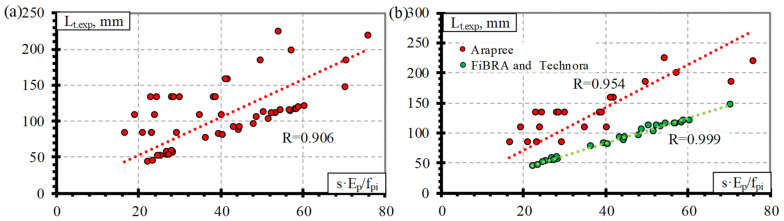
Comparison between the transfer length of the AFRP bars and s·E_p_/f_pi_: (**a**) all results, (**b**) effect of the type of reinforcement.

**Figure 7:**
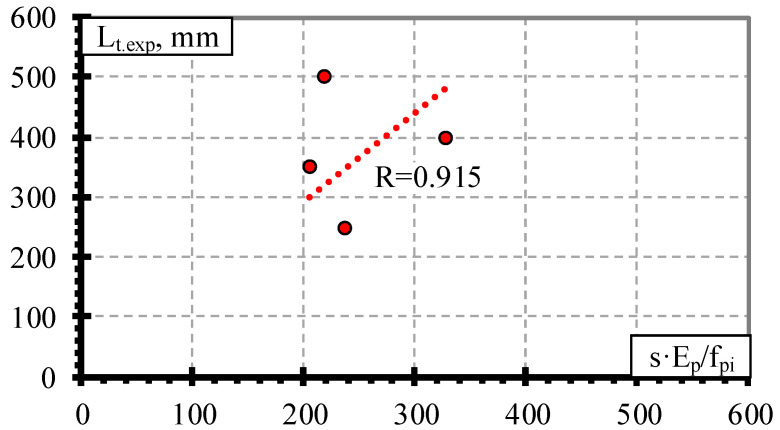
Comparison between the transfer length of the BFRP bars and s·E_p_/f_pi_.

Figure 4, Figure 5, Figure 6 and Figure 7 show the relationship between the transfer length of different FRP reinforcements (CFCC, CFRP, AFRP, and BFRP) and s·E_p_/f_pi_. Therefore, the distribution of the results in these figures represents the proposed average values of the bond shape factor α.

Comparison of the transfer length of the CFCC seven-wire strand and s·E_p_/f_pi_ shows the distribution of the experimental results with an average value of the bond shape factor α = 3.45 with STD = 0.78, COV = 22.6%, and R^2^ = 0.96 (Table 4 and Figure 4a) (for concrete strength of 23–48 MPa, prestress level 0.49–0.81%, and reinforcement diameter 10.5–15.2 mm). Figure 4b shows that the type of release (gradual or sudden) of prestressed reinforcement can influence the transfer length of the CFCC strands. In [10], it was concluded that there is a clear difference in the transfer length of the prestressed CFCC strands affected by the sudden or gradual type of release. However, the database of transfer length and slip results of CFCC strands (Table A1) affected by a sudden transfer of prestressing consists only of three specimens. Therefore, the number of specimens tested is not sufficient, and the database should be increased with additional research results to draw solid conclusions.

Figure 5a presents the relationship between the transfer length of CFRP bars and s·E_p_/f_pi_, with an average value of the bond shape factor α = 2.54 with STD = 0.50, COV = 19.6%, and R^2^ = 0.98 (Table 4) (for concrete strength of 26–50.7 MPa, prestress level 0.33–0.86%, and reinforcement diameter 7.9–12.7 mm). Two types of CFRP bars are evaluated in the database: CFRP Leadline bars with a spirally indented surface and CFRP bars with the sand-coated surface. According to Figure 5b, it can be seen that the transfer length of the CFRP Leadline bar and CFRP bar is not influenced by the reinforcement surface conditions. Therefore, it can be concluded that the spirally indented and sand-coated CFRP reinforcement has similar bond conditions. According to the trend lines in Figure 5b, there is a small influence of the shear reinforcement and the type of reinforcement release (sudden or gradual). However, the distribution of the results shows that it is almost negligible. The same conclusions were made in [10].

Comparison of the transfer length of the AFRP bars (Arapree, FiBRA, and Technora) and s·E_p_/f_pi_ shows the distribution of the experimental results with an average value of the bond shape factor α = 2.94 with STD = 1.15, COV = 39.2%, and R^2^ = 0.90 (Table 4 and Figure 6a) (for concrete strength of 54.6–81.5 MPa, prestress level 0.50–0.67%, and reinforcement diameter 3.7–7.5 mm). The surface of the AFRP Arapree, FiBRA, and Technora bars was sand-coated, braided, and spirally wounded, respectively. Therefore, Figure 6b shows the influence of different surface conditions on the transfer length of the AFRP bars. It can be seen that the results of the AFRP FiBRA and Technora bars are similar; therefore, the value of the bond shape factor is α = 2.1 with STD = 0.07, COV = 3.37%, and R^2^ = 0.95 (Table 4) (for concrete strength of 56–58 MPa, prestress level 0.59–0.67%, and reinforcement diameter 3.7–4.0 mm). The results of AFRP Arapree bars differ from those of the AFRP FiBRA and Technora bars. Therefore, α = 4.04 for Arapree bars with STD = 0.95, COV = 23.5%, and R^2^ = 0.99 (Table 4) (for concrete strength of 56.4–81.5 MPa, prestress level 0.50%, and reinforcement diameter 5.3–7.5 mm). It should be mentioned that the results of [21] from the database for AFRP Arapree bars were determined with a wider range of concrete strength (54.6–81.5 MPa) and reinforcement diameter (5.3–7.5 mm) compared to the results of [22] for FiBRA and Technora bars with small or no variation of initial parameters. However, the division of the results according to reinforcement type reduced the variation of the results from 39.2% (all results) to 3.37% for FiBRA and Technora bars and 23.5% for Arapree bars.

The results of the BFRP bar database (Table A4 in Appendix A) showed that the comparison of the transfer length and s·E_p_/f_pi_ gave an average value of the bond shape factor α = 1.57 with STD = 0.48, COV = 30.6%, and R^2^ = 0.91 (Table 4 and Figure 7) (for concrete strength of 27 MPa, prestress level 0.31–0.34%, and reinforcement diameter 8.0 mm). The literature review showed that Crosset et al. [43] tested only two beams prestressed with BFRP bars to determine the transfer length. However, it does not provide clear information on the slip of BFRP bars at the end of the specimen at transfer corresponding to the transfer length. Additionally, Motwani et al. [23] have tested four beams and determined the transfer length and corresponding slip of prestressed BFRP bars. Therefore, the results of only four specimens were analyzed in this research. Additionally, the initial parameters were almost identical for all four specimens (Table 3), and it is not sufficient to draw strong conclusions.

### 4.4. Comparison of Experimental and Theoretical Results

This section presents a comparison of experimental and theoretical results of transfer length of different types (CFCC, CFRP, AFRP, and BFRP) of FRP reinforcement. Theoretical results are based on Equations (1), (3)–(5) provided in Table 1. Equation (2) is not taken into account because it is the same as Equation (1) only with a slightly different value of α = 2.08. Equation (6) is also not evaluated due to the small number of influencing parameters. It only evaluates the slip(s) and empirical coefficient (K) which was derived for a 12.7 mm diameter seven-wire steel strand. It was stated that in the case of constant bond stress distribution α = 2 and in the case of linear bond stress distribution α = 3 in Equation (1). Therefore, the comparison of the experimental transfer length results determined according to Equation (1) is carried out for both suggested extreme values (2 and 3) of the bond shape factor (α).

**Figure 8:**
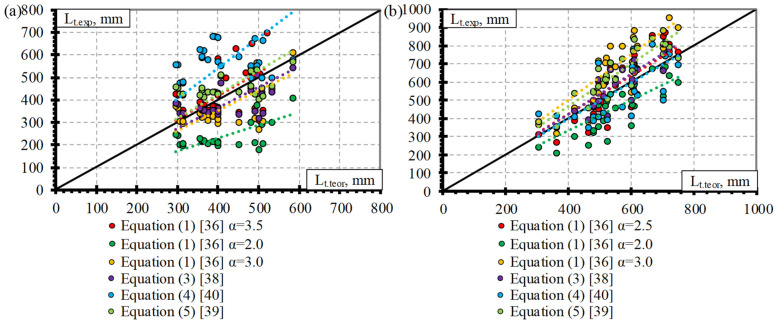
Comparison of experimental and theoretical transfer length results of (**a**) CFCC strands and (**b**) CFRP bars.

Figure 8a presents a comparison of the experimental and theoretical transfer length results of CFCC strands. It can be seen that the theoretical prediction of the transfer length according to Equation (4) overestimates the experimental results on average by 45% (L_t.teor_/L_t.exp_ = 1.45) with STD = 0.29 and COV = 19.8%, and the results with α = 2 (Equation (1)) underestimates the experimental results on average by 39% (L_t.teor_/L_t.exp_ = 0.61) with STD = 0.13 and COV = 20.8%. However, the theoretical results determined with α = 3 (Equation (1)) and according to Equation (3) have a lower underestimation of the experimental results with L_t.teor_/L_t.exp_ = 0.91, STD = 0.19, and COV = 20.8% and with L_t.teor_/L_t.exp_ = 0.95 STD = 0.17 and COV = 17.5%, respectively. Equation (5) proposed by [39] gave a close prediction of the experimental results with an average overestimation of 13% (L_t.teor_/L_t.exp_ = 1.13) with STD = 0.20 and COV = 18.1%. However, the most accurate prediction of the experimental transfer length results of the CFCC strands was determined by Equation (1) with the proposed α = 3.45 (Table 4) with L_t.teor_/L_t.exp_ = 1.05, STD = 0.22, and COV = 20.8%, which is on the safe side.

Figure 8b provides a comparison of the experimental and theoretical prediction of the transfer length of CFRP bars. The introduction of α = 2 and α = 3 in Equation (1) gave the most inaccurate prediction of the experimental results with L_t.teor_/L_t.exp_ = 0.78, STD = 0.20, and COV = 26%, and with L_t.teor_/L_t.exp_ = 1.18 STD = 0.31 and COV = 26%, respectively. The relationship proposed by [39] (Equation (5)) showed an overestimation of the experimental results on an average of 13% (L_t.teor_/L_t.exp_ = 1.13) with STD = 0.25 and COV = 21.9%, and Equation (4) proposed by [40] showed good agreement with the experimental results with L_t.teor_/L_t.exp_ = 0.97, STD = 0.28 and COV = 29%. The theoretical transfer length results with the proposed α = 2.54 (Equation (1)) for CFRP bars and determined according to Equation (3) proposed by [38] were in close agreement with the experimental results with L_t.teor_/L_t.exp_ = 1.03, STD = 0.18, and COV = 17.2%, and with L_t.teor_/L_t.exp_ = 1.03, STD = 0.23, and COV = 22.8%, respectively. The relationships proposed by [40] (Equation (4)), [38] (Equation (3)), and [36] (Equation (1)) were in good agreement between the theoretical and experimental results with a difference of only 3%. However, the results of Equation (4), on average, are not on the safe side, and the variation of the results is greater (COV = 29%) compared to the results (COV = 17.2%) determined according to Equation (1) with α = 2.54. The variation in the theoretical results predicted according to Equation (3) is also higher (COV = 22.8%) compared to the results ((COV = 17.2%) predicted according to Equation (1) with α = 2.54.

**Figure 9:**
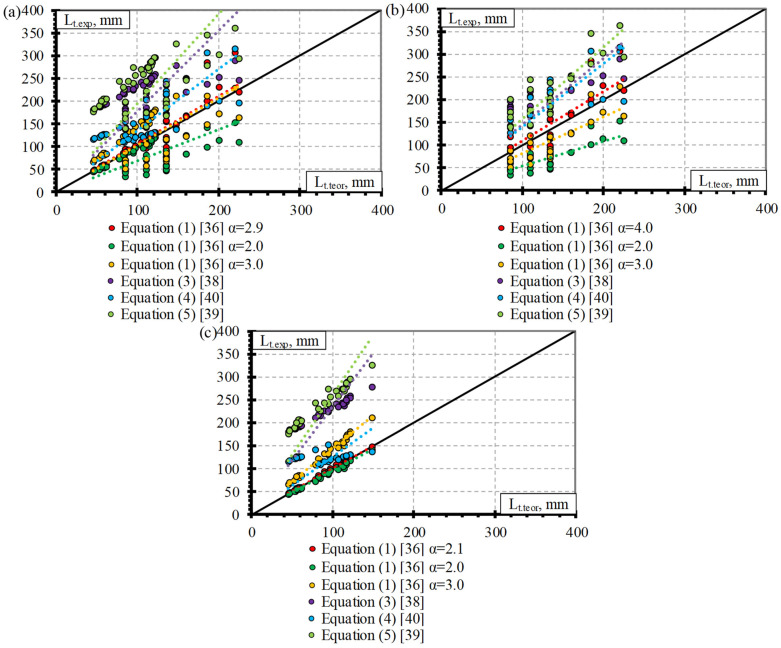
Comparison of experimental and theoretical transfer length results of AFRP bars with different surface properties: (**a**) all results, (**b**) Arapree, (**c**) FiBRA and Technora.

As presented in Section 4.3, the AFRP bars were divided into two groups according to the surface properties and the type of reinforcement. Therefore, the comparison of the experimental and theoretical transfer length results of the AFRP bars is presented for three situations: for all AFRP bars (Figure 9a), for the AFRP Arapree bars (Figure 9b), and for the AFRP FiBRA and Technora bars (Figure 9c).

The overestimation of the experimental transfer length results of all types of AFRP bars is predicted according to Equation (5), Equation (3), and Equation (4) on average with L_t.teor_/L_t.exp_ = 2.36, STD = 0.76, and COV = 32.4%, with L_t.teor_/L_t.exp_ = 2.22, STD = 0.74, and COV = 33.5%, and with L_t.teor_/L_t.exp_ = 1.53, STD = 0.44, and COV = 28.8%, respectively (Figure 9a). The theoretical results predicted with α = 2 underestimated the experimental results by 23% (L_t.teor_/L_t.exp_ = 0.77) with STD = 0.23, and COV = 30.3%. The proposed value of the bond shape factor for all types of AFRP bars presented in the database (Table A3 in Appendix A) is α = 2.94 and is almost equal to the value of α = 3 proposed by [36] in the case of linear bond stress distribution. Therefore, the overestimation of the experimental transfer length results was 13–15% (L_t.teor_/L_t.exp_ = 1.13–1.15) with STD = 0.34–0.35 and COV = 30.3%.

The results of the AFRP Arapree bar database are compared with the theoretically predicted transfer length in Figure 9b. It is evident that the theoretical results determined according to Equation (5), Equation (3), and Equation (4) show an overestimation of the experimental transfer length results on average with L_t.teor_/L_t.exp_ = 1.66, STD = 0.30, and COV = 18.2%, with L_t.teor_/L_t.exp_ = 1.62, STD = 0.34, and COV = 20.8%, and with L_t.teor_/L_t.exp_ = 1.50, STD = 0.25, and COV = 16.5%, respectively. However, the use of α = 2 and α = 3 in Equation (1) shows an underestimation of the experimental results on average with L_t.teor_/L_t.exp_ = 0.52, STD = 0.12, and COV = 22.7%, and with L_t.teor_/L_t.exp_ = 0.78, STD = 0.18, and COV = 22.7%, respectively. Therefore, the best prediction of the experimental and theoretical results is determined with a proposed value of α = 4 (Figure 9b) for the AFRP Arapree bars. The theoretical results were 5% higher (L_t.teor_/L_t.exp_ = 1.05) than the experimental results with STD = 0.25 and COV = 23.2%.

The most inaccurate prediction of the experimental transfer length results of the AFRP FiBRA and Technora bars was determined according to Equation (5) (L_t.teor_/L_t.exp_ = 2.89, STD = 0.54, and COV = 18.7%) and Equation (3) (L_t.teor_/L_t.exp_ = 2.67, STD = 0.63, and COV = 23.6%) (Figure 9c). The overestimation of the experimental transfer length results was lower according to Equation (1) with α = 3 and Equation (4) with L_t.teor_/L_t.exp_ = 1.43, STD = 0.05, and COV = 3.32% and L_t.teor_/L_t.exp_ = 1.55, STD = 0.54, and COV = 35.2%, respectively. The theoretical results with α = 2 gave close agreement with the experimental results. However, the experimental results were overestimated by 5% (L_t.teor_/L_t.exp_ = 1.05) with STD = 0.03 and COV = 3.32%. The best fit of the experimental and theoretical transfer length results of the AFRP FiBRA and Technora bars was achieved with the proposed value of the bond shape factor α = 2.1 on average with L_t.teor_/L_t.exp_ = 1.00, STD = 0.03, and COV = 3.32%.

**Figure 10:**
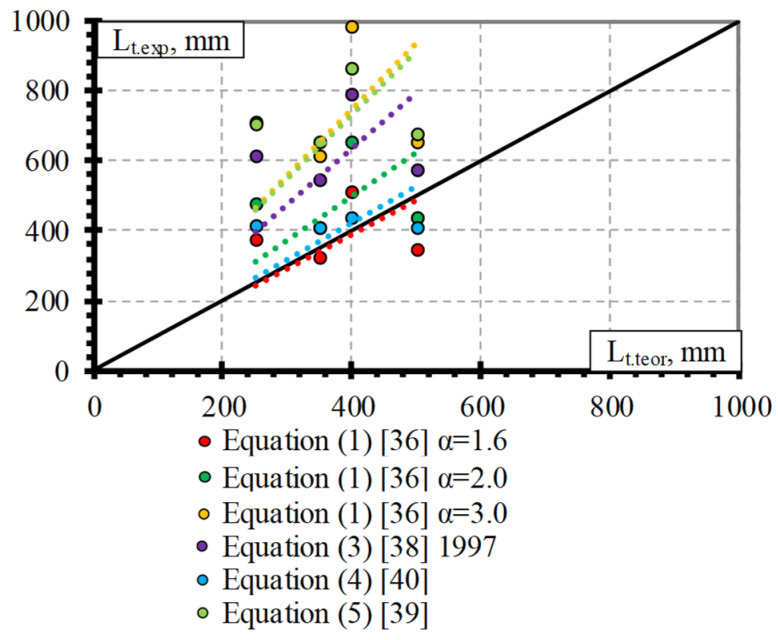
Comparison of experimental and theoretical transfer length results of the BFRP bars.

The theoretical and experimental prediction of the transfer length results of the BFFP bars is presented in Figure 10. The overestimation of the experimental results is predicted with α = 3 (Equation (1)), according to Equation (5), Equation (3), with α = 2 (Equation (1)), and according to Equation (4) on average with L_t.teor_/L_t.exp_ = 2.09, STD = 0.69, and COV = 32.9%, with L_t.teor_/L_t.exp_ = 2.05, STD = 0.61, and COV = 29.9%, with L_t.teor_/L_t.exp_ = 1.78, STD = 0.56, and COV = 31.2%, with L_t.teor_/L_t.exp_ = 1.40, STD = 0.46, and COV = 32.9%, and with L_t.teor_/L_t.exp_ = 1.18, STD = 0.35, and COV = 29.3%, respectively. The proposed value of the bond shape factor α = 1.57 gave a significantly lower overestimation of the experimental transfer length results of the BFRP bars on average with L_t.teor_/L_t.exp_ = 1.09, STD = 0.36, and COV = 32.9%.

**Figure 11:**
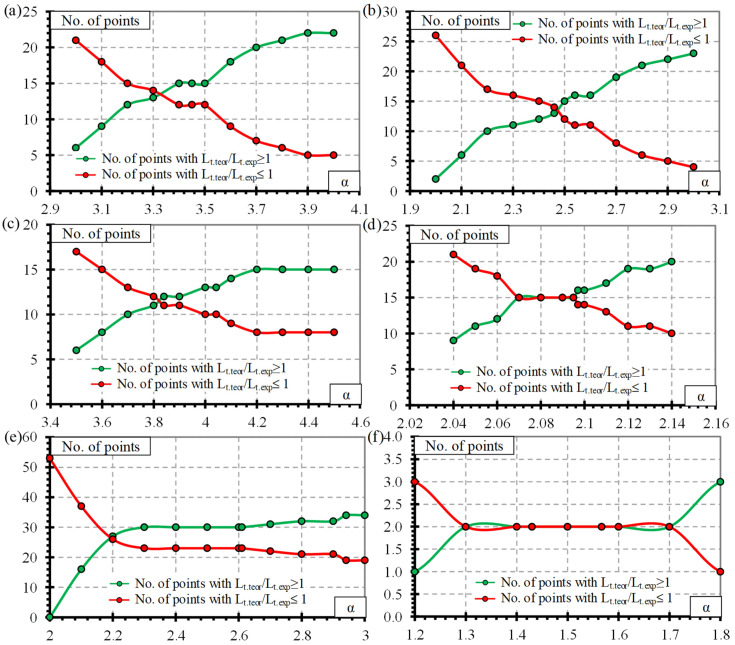
Comparison between the coefficient α and the number of experimental points of (**a**) CFCC strands, (**b**) CFRP bars, (**c**) AFRP Arapree bars, (**d**) AFRP Fibra and Technora bars, (**e**) all AFRP bars and (**f**) BFRP bars.

Statistical analysis was additionally performed to verify the proposed values of the bond shape factor α. The statistical results for the CFCC strands (Table A5), CFRP bars (Table A6), AFRP Arapree bars (Table A7), AFRP Fibra and Technora bars (Table A8), all AFRP bars (Table A9), and BFRP bars (Table A10) are presented in Appendix B. The analyzed results are M, STD and COV—mean value, standard deviation, and coefficient of variation of L_t.teor_/L_t.exp_, respectively, N_total_—total number of specimens, OV and UV—overestimated and underestimated values of L_t.teor_/L_t.exp_, respectively. The comparison of the bond shape factor α and a number of experimental points overestimating (OV = L_t.teor_/L_t.exp_ ≥ 1) and underestimating (UV = L_t.teor_/L_t.exp_ ≤ 1) the experimental transfer length results of different FRP reinforcements are provided in Figure 11.

It can be seen in Table A5 that the mean value of L_t.teor_/L_t.exp_ = 1 for CFCC strands when the bond shape factor α = 3.3. Furthermore, it is confirmed in Figure 11a (OV = 13 and UV = 14 are almost equal). However, the proposed α = 3.45 gives an average overestimation of the experimental results by 5% with OV = 15 and UV = 12, which is on the safe side compared to the results with α = 3.3. Additionally, the same values of OV = 15 and UV = 12 are valid when α = 3.4…3.5 with L_t.teor_/L_t.exp_ = 1.03…1.06.

The results of CFRP bars (Table A6 and Figure 11b) show that α = 2.46 gives L_t.teor_/L_t.exp_ = 1 with OV = 13 and UV = 14, and the proposed value α = 2.5 gives L_t.teor_/L_t.exp_ = 1.02 with OV = 15 and UV = 12. It shows that the results are quite sensitive to the change of α and therefore more points are close to the perfect prediction of the experimental transfer length (L_t.teor_/L_t.exp_ = 1).

The statistical results of the AFRP Arapree bars (Table A7 and Figure 11c) show that the best average fit (L_t.teor_/L_t.exp_ = 1) of the experimental results was obtained with α = 3.84 (OV = 12 and UV = 11). However, the results with the proposed α = 4.0 are on the safe side on average by 4% with higher OV = 13 and lower UV = 10.

Statistical analysis of AFRP Fibra and Technora bars (Table A8 and Figure 11d) showed that the results are very sensitive to changes in bond shape factor α. It can be related to the low COV = 3.3% of the L_t.teor_/L_t.exp_ results. The proposed value of α = 2.1 coincides with the statistical results with L_t.teor_/L_t.exp_ = 1, OV = 16, and UV = 14.

The best fit of all results of the AFRP bars (L_t.teor_/L_t.exp_ = 1) is obtained with α = 2.61 with OV = 30 and UV = 23 (Table A9). However, α < 2.61 gives an average underestimation, and α > 2.61 gives an average overestimation of the experimental results. The bond shape factor α between 2.3 and 2.9 gives similar OV = 30…32 and UV = 21…23 with L_t.teor_/L_t.exp_ = 0.88…1.11. Therefore, the results are insensitive to the variation of the bond shape factor α. Furthermore, it can be seen that AFRP reinforcement should be evaluated according to the type of reinforcement (Arapree, Fibra, Technora, etc.).

The database of transfer length versus slip of different types of FRP reinforcement (CFCC–27, CFRP–27, AFRP Arapree–23, AFRP Fibra and Technora–30, and BFRP–4) is still not sufficient to draw solid conclusions suggesting the use of the proposed bond shape factors α inpractice. The proposed values of α = 3.5 for CFCC strands, α = 2.5 for CFRP bars, α = 4.0 for AFRP Arapree bars, and α = 2.1 for AFRP Fibra and Technora bars give a slight overestimation of the experimental transfer length results, which is on the safe side.

## 5. Conclusions

The analysis of the collected database of transfer length and slip of different FRP reinforcements together with the analysis of theoretical models led to the following conclusions and proposals:

1. From the review of methods for the determination of the transfer length of prestressed reinforcement, it is evident that each of the methods has advantages and disadvantages. The most accurate method to determine the transfer length of prestressed reinforcement is to measure the strains within the reinforcement. The most unreliable method is to measure the strains on the surface of reinforcement. However, the easiest and most straightforward method with sufficient accuracy is related to the slip of reinforcement;
2. After the analysis of the results of a larger database, new bond shape factors α were proposed for the relationship between the transfer length and the slip of different FRP reinforcements. The proposed value for the CFCC strands is α = 3.5 (for concrete strength of 23–48 MPa, prestress level 0.49–0.81%, and reinforcement diameter 10.5–15.2 mm) and for CFRP bars is α = 2.5 (for concrete strength of 26–50.7 MPa, prestress level 0.33–0.86%, and reinforcement diameter 7.9–12.7 mm).
3. It was determined that there is a correlation between the type of reinforcement (surface conditions) and the transfer length of the AFRP bars. Therefore, new values of α are proposed: α = 2.1 for AFRP FiBRA and Technora bars (for concrete strength of 56–58 MPa, prestress level 0.59–0.67%, and reinforcement diameter 3.7–4.0 mm) and α = 4.0 for AFRP Arapree bars (for concrete strength of 56.4–81.5 MPa, prestress level 0.50%, and reinforcement diameter 5.3–7.5 mm);
4. The comparison of experimental and theoretical results showed that the theoretical models derived for steel strands in some cases can predict the transfer length of FRP reinforcement. However, different models showed a close prediction of the experimental results of different types of FRP reinforcement with no consistency. Therefore, Equations (3)–(5) are not sufficiently adequate to predict the transfer length of pretensioned FRP reinforcement;
5. Equation (1) gives the most accurate prediction of the transfer length of different FRP reinforcements with the proposed bond shape factors α. Therefore, it can be applied not only to steel strands but also to prestressed FRP reinforcement with sufficient accuracy;
6. Analysis of the relationship between transfer length and slip of FRP reinforcement during transfer together with the proposed new values of bond shape factor α provides possibilities to adopt the slippage monitoring method in the production and quality control of precast and prestressed concrete structures and perform additional research to increase the understanding of the transfer length of FRP reinforcement. In particular, more attention should be given to the release type of prestressed FRP reinforcement. Furthermore, the influence of the surface conditions of AFRP and CFRP reinforcement should be analyzed. The database should be increased with a wider range of initial variables (f_ci_, f_pi_/f_pu_, Ø, c) for the transfer length of different types of AFRP reinforcement. Furthermore, additional research on the transfer length of BFRP reinforcement should be performed by analyzing the influence of the prestress transfer method, different surface conditions, and other important parameters (f_ci_, f_pi_/f_pu_, c).

## Figures and Tables

**Table 2:** Summary of the bond shape factor α.

Reference	Reinforcement Type	α
[43]	BFRP sand coated bar	2.82–3.32
[12]	CFRP Leadline bar	2.91
	CFCC strand	2.48
[21]	AFRP Arapree sand coated bar	3.03

**Table 3:** Initial parameters of the transfer length versus slip.

FRP Type	c, mm	Ø, mm	c/Ø	A_p_, mm^2^	E_p_, GPa	f_pu_, MPa	f_pi_, MPa	f_pi_/f_pu_	f_ci_, MPa
CFCC	38.2–60	10.5–15.2	3.1–4.3	55.7–113.6	138–141	1725–1889	913–1408	0.49–0.81	23–48
CFRP	31.8–40.5	7.9–12.7	2.5–5.1	46.1–126.7	124.1–147	1765–2275	634–1930	0.33–0.86	26–50.7
AFRP	12.4–68	3.7–7.5	2.3–17	11–22.2	54–91	1427–3000	836–1486	0.50–0.67	54.6–81.5
BFRP	46.0	8.0	5.7	50.2	44	1126	369–381	0.31–0.34	27

**Table 4:** Summary of the bond shape factor α.

α	CFCC	CFRP	AFRP			BFRP
			All	Arapree	FiBRA	
Mean	3.45	2.54	2.94	4.04	2.1	1.57
STD	0.78	0.50	1.15	0.95	0.07	0.48
COV, %	22.6	19.6	39.2	23.5	3.37	30.6

## Data Availability

Not applicable.

## Appendix A

**Table A1:** Experimental parameters of specimens with pretensioned CFCC reinforcement.

Reference	Specimen No	FRP Type	FRP Surface	Specimen Type	Release Type	Shear Reinforcement	Specimen Dimensions (b × h × l, mm)	c, mm	Ø, mm	A_p_, mm^2^	E_p_, GPa	f_pu_, MPa	f_pi_, MPa	f_pi_/f_pu_	f_ci_, MPa	L_t_, mm	Slip, mm
[12,15]	BT11	CFCC 7-wire Strand	Helical plain	Beam	Gradual	Yes	80 × 250 × 1380	45.0	10.5	55.7	140	1725	1400	0.81	29.0	305.0	1.01
	BT12			Beam	Gradual	Yes	80 × 250 × 1381	45.0	10.5	55.7	140	1725	1400	0.81	29.0	312.5	0.99
	BT13			Beam	Gradual	Yes	80 × 250 × 1382	45.0	10.5	55.7	140	1725	1400	0.81	29.0	312.5	1.02
	BT14			Beam	Gradual	Yes	80×250×1383	45.0	10.5	55.7	140	1725	1400	0.81	29.0	312.5	1.02
	BT7			Beam	Gradual	Yes	100×250×1380	50.0	12.5	76.0	141	1868	1408	0.75	28.0	400.0	0.99
	BT8			Beam	Gradual	Yes	100 × 250 × 1381	50.0	12.5	76.0	141	1868	1408	0.75	28.0	375.0	1.03
	BT9			Beam	Gradual	Yes	100 × 250 × 1382	50.0	12.5	76.0	141	1868	1408	0.75	28.0	360.0	1.08
	BT10			Beam	Gradual	Yes	100 × 250 × 1383	50.0	12.5	76.0	141	1868	1408	0.75	28.0	360.0	1.1
	BT15			Beam	Gradual	Yes	100 × 250 × 2750	50.0	12.5	76.0	141	1868	1125	0.60	30.0	300.0	0.97
	BT16			Beam	Gradual	Yes	100 × 250 × 2751	50.0	12.5	76.0	141	1868	1125	0.60	30.0	295.0	0.99
	BT19			Beam	Gradual	No	175 × 250 × 2750	50.0	12.5	76.0	141	1868	1355	0.73	23.0	500.0	0.86
	BT20			Beam	Gradual	No	175×250×2750	50.0	12.5	76.0	141	1868	1355	0.73	23.0	450.0	0.96
	BT1			Beam	Gradual	Yes	120 × 300 × 1720	60.0	15.2	113.6	138	1750	1074	0.61	35.0	365.0	0.85
	BT2			Beam	Gradual	Yes	120 × 300 × 1720	60.0	15.2	113.6	138	1750	1074	0.61	35.0	355.0	0.88
	BT3			Beam	Gradual	Yes	120 × 300 × 1720	60.0	15.2	113.6	138	1750	1312	0.75	31.0	392.5	1.03
	BT4			Beam	Gradual	Yes	120 × 300 × 1721	60.0	15.2	113.6	138	1750	1312	0.75	31.0	387.5	1.03
	BT5			Beam	Gradual	Yes	120 × 300 × 1722	60.0	15.2	113.6	138	1750	1312	0.75	31.0	400.0	1.01
	BT6			Beam	Gradual	Yes	120 × 300 × 1723	60.0	15.2	113.6	138	1750	1312	0.75	31.0	400.0	1
	BT17			Beam	Gradual	No	200 × 300 × 3500	60.0	15.2	113.6	138	1750	1294	0.74	22.0	650.0	0.82
	BT18			Beam	Gradual	No	200 × 300 × 3500	60.0	15.2	113.6	138	1750	1294	0.74	22.0	600.0	0.89
	BT21			Beam	Gradual	No	175 × 300 × 3500	60.0	15.2	113.6	138	1750	1083	0.62	24.0	510.0	0.8
	BT22			Beam	Gradual	No	175 × 300 × 3500	60.0	15.2	113.6	138	1750	1083	0.62	24.0	490.0	0.83
[17]	CDT2-1-WA-A1	CFCC 7-wire Strand	Helical plain	Girder	Sudden	Yes	Double T	38.2	12.5	76.0	138	1868	925	0.50	48.0	501.5	1
	CDT2-1-WA-A2			Girder	Sudden	Yes	Double T	38.2	12.5	76.0	138	1869	901	0.48	48.0	-	2
	CDT2-1-WA-A3			Girder	Sudden	Yes	Double T	38.2	12.5	76.0	138	1870	878	0.47	48.0	-	2.3
	CDT2-1-WA-B1			Girder	Sudden	Yes	Double T	38.2	12.5	76.0	138	1871	954	0.51	48.0	419.0	1
	CDT2-1-WA-B2			Girder	Sudden	Yes	Double T	38.2	12.5	76.0	138	1872	954	0.51	48.0	-	0.3
	CDT2-1-WA-B3			Girder	Sudden	Yes	Double T	38.2	12.5	76.0	138	1873	913	0.49	48.0	-	0.8
	CDT2-2-WA-A1			Girder	Sudden	Yes	Double T	38.2	12.5	76.0	138	1874	954	0.51	48.0	482.5	1.3
	CDT2-2-WA-A2			Girder	Sudden	Yes	Double T	38.2	12.5	76.0	138	1875	959	0.51	48.0	-	1
	CDT2-2-WA-A3			Girder	Sudden	Yes	Double T	38.2	12.5	76.0	138	1876	942	0.50	48.0	-	1.8
	CDT2-2-WA-B1			Girder	Sudden	Yes	Double T	38.2	12.5	76.0	138	1877	913	0.49	48.0	468.5	1
	CDT2-2-WA-B2			Girder	Sudden	Yes	Double T	38.2	12.5	76.0	138	1878	924	0.49	48.0	-	1.3
	CDT2-2-WA-B3			Girder	Sudden	Yes	Double T	38.2	12.5	76.0	138	1879	977	0.52	48.0	-	0.5
	CDT2-3-WA-A1			Girder	Sudden	Yes	Double T	38.2	12.5	76.0	138	1880	1018	0.54	48.0	520.5	1.5
	CDT2-3-WA-A2			Girder	Sudden	Yes	Double T	38.2	12.5	76.0	138	1881	966	0.51	48.0	-	1
	CDT2-3-WA-A3			Girder	Sudden	Yes	Double T	38.2	12.5	76.0	138	1882	983	0.52	48.0	-	0.5
	CDT2-3-WA-B1			Girder	Sudden	Yes	Double T	38.2	12.5	76.0	138	1883	972	0.52	48.0	412.5	1.5
	CDT2-3-WA-B2			Girder	Sudden	Yes	Double T	38.2	12.5	76.0	138	1884	1001	0.53	48.0	-	1.3
	CDT2-3-WA-B3			Girder	Sudden	Yes	Double T	38.2	12.5	76.0	138	1885	1036	0.55	48.0	-	0.8
	CDT2-4-WA-A1			Girder	Sudden	Yes	Double T	38.2	12.5	76.0	138	1886	983	0.52	48.0	443.0	1.3
	CDT2-4-WA-A2			Girder	Sudden	Yes	Double T	38.2	12.5	76.0	138	1887	1095	0.58	48.0	-	1
	CDT2-4-WA-A3			Girder	Sudden	Yes	Double T	38.2	12.5	76.0	138	1888	995	0.53	48.0	-	0.8
	CDT2-4-WA-B1			Girder	Sudden	Yes	Double T	38.2	12.5	76.0	138	1889	1059	0.56	48.0	400.0	1.3
	CDT2-4-WA-B2			Girder	Sudden	Yes	Double T	38.2	12.5	76.0	138	1890	1024	0.54	48.0	-	1.3
	CDT2-4-WA-B3			Girder	Sudden	Yes	Double T	38.2	12.5	76.0	138	1891	1240	0.66	48.0	-	1.3

**Table A2:** Experimental parameters of specimens with pretensioned CFRP reinforcement.

Reference	Specimen No	FRP Type	FRP Surface	Specimen Type	Release Type	Shear Reinforcement	Specimen Dimensions (b × h × l, mm)	c, mm	Ø, mm	A_p_, mm^2^	E_p_, GPa	f_pu_, MPa	f_pi_, MPa	f_pi_/f_pu_	f_ci_, MPa	L_t_, mm	Slip, mm
[18]	B1-4-65	CFRP Leadline Bar	Spirally Indented Sanded	Beam	Gradual	Yes	139.7 × 247.7 × 4000	31.8	12.7	126.5	124	2275	1345	0.59	45.4	607.1	3.07
	B2-4-55			Beam	Gradual	Yes	139.7 × 247.7 × 4001	31.8	12.7	126.5	124	2275	1138	0.50	47.9	533.4	2.44
	B3-4-60			Beam	Gradual	Yes	139.7 × 247.7 × 4002	31.8	12.7	126.5	124	2275	1241	0.55	46.8	495.3	2.39
	B4-4-60			Beam	Gradual	Yes	139.7 × 247.7 × 4003	31.8	12.7	126.5	124	2275	1282	0.56	50.7	342.9	3.07
[12,15]	BL1	CFRP Leadline Bar	Spirally Indented	Beam	Gradual	Yes	80 × 200 × 1700	35.0	8.0	46.1	147	1970	1193	0.61	34.0	452.5	0.88
	BL2			Beam	Gradual	Yes	80 × 200 × 1701	35.0	8.0	46.1	147	1970	1193	0.61	34.0	462.5	1.04
	BL3			Beam	Gradual	Yes	80 × 200 × 1702	35.0	8.0	46.1	147	1970	1410	0.72	35.0	480.0	1.56
	BL4			Beam	Gradual	Yes	80 × 200 × 1703	35.0	8.0	46.1	147	1970	1410	0.72	35.0	480.0	1.12
	BL5			Beam	Gradual	Yes	80 × 250 × 2500	35.0	8.0	46.1	147	1970	1497	0.76	28.0	620.0	2.71
	BL6			Beam	Gradual	Yes	80 × 250 × 1700	35.0	8.0	46.1	147	1970	1497	0.76	28.0	625.0	-
	BL7			Beam	Gradual	Yes	80 × 250 × 2100	35.0	8.0	46.1	147	1970	1497	0.76	28.0	610.0	3
	BL8			Beam	Gradual	Yes	80 × 250 × 2100	35.0	8.0	46.1	147	1970	1497	0.76	28.0	625.0	-
	BL9			Beam	Gradual	No	150 × 250 × 4000	36.0	8.0	46.1	147	1970	1258	0.64	26.0	700.0	2.25
	BL10			Beam	Gradual	No	150 × 250 × 4000	36.0	8.0	46.1	147	1970	1258	0.64	26.0	700.0	2.88
	BL11			Beam	Gradual	No	150 × 250 × 3900	36.0	8.0	46.1	147	1970	1518	0.77	42.0	600.0	1.88
	BL12			Beam	Gradual	No	150 × 250 × 3900	36.0	8.0	46.1	147	1970	1518	0.77	42.0	500.0	1.18
	PL1			Prism	Gradual	No	70 × 95 × 1884	35.0	8.0	46.1	147	1970	1193	0.61	34.0	490.0	0.73
	PL2			Prism	Gradual	No	70 × 95 × 1884	35.0	8.0	46.1	147	1970	1193	0.61	34.0	480.0	1.36
	PL3			Prism	Gradual	No	80 × 80 × 1884	40.0	8.0	46.1	147	1970	1410	0.72	31.0	540.0	0.97
	PL4			Prism	Gradual	No	80 × 80 × 1884	40.0	8.0	46.1	147	1970	1410	0.72	31.0	525.0	1.33
[17]	CDT1-1-WA-A1	CFRP Leadline Bar	Spirally Indented	Girder	Sudden	Yes	Double T	40.5	7.9	46.1	147	2256	1740	0.77	48.0	557.5	5.2
	CDT1-1-WA-A2			Girder	Sudden	Yes	Double T	40.5	7.9	46.1	147	2256	1830	0.81	48.0	-	-
	CDT1-1-WA-A3			Girder	Sudden	Yes	Double T	40.5	7.9	46.1	147	2256	1300	0.58	48.0	-	2.2
	CDT1-1-WA-B1			Girder	Sudden	Yes	Double T	40.5	7.9	46.1	147	2256	1540	0.68	48.0	507.5	2.8
	CDT1-1-WA-B2			Girder	Sudden	Yes	Double T	40.5	7.9	46.1	147	2256	1540	0.68	48.0	-	-
	CDT1-1-WA-B3			Girder	Sudden	Yes	Double T	40.5	7.9	46.1	147	2256	1360	0.60	48.0	-	-
	CDT1-2-WA-A1			Girder	Sudden	Yes	Double T	40.5	7.9	46.1	147	2256	1640	0.73	48.0	470.0	-
	CDT1-2-WA-A2			Girder	Sudden	Yes	Double T	40.5	7.9	46.1	147	2256	1740	0.77	48.0	-	-
	CDT1-2-WA-A3			Girder	Sudden	Yes	Double T	40.5	7.9	46.1	147	2256	1120	0.50	48.0	-	-
	CDT1-2-WA-B1			Girder	Sudden	Yes	Double T	40.5	7.9	46.1	147	2256	1540	0.68	48.0	-	-
	CDT1-2-WA-B2			Girder	Sudden	Yes	Double T	40.5	7.9	46.1	147	2256	1640	0.73	48.0	-	-
	CDT1-2-WA-B3			Girder	Sudden	Yes	Double T	40.5	7.9	46.1	147	2256	1120	0.50	48.0	-	-
	CDT1-3-WA-A1			Girder	Sudden	Yes	Double T	40.5	7.9	46.1	147	2256	1640	0.73	48.0	502.5	7.9
	CDT1-3-WA-A2			Girder	Sudden	Yes	Double T	40.5	7.9	46.1	147	2256	1640	0.73	48.0	-	3.3
	CDT1-3-WA-A3			Girder	Sudden	Yes	Double T	40.5	7.9	46.1	147	2256	1050	0.47	48.0	-	0.8
	CDT1-3-WA-B1			Girder	Sudden	Yes	Double T	40.5	7.9	46.1	147	2256	1930	0.86	48.0	415.0	2
	CDT1-3-WA-B2			Girder	Sudden	Yes	Double T	40.5	7.9	46.1	147	2256	1540	0.68	48.0	-	1
	CDT1-3-WA-B3			Girder	Sudden	Yes	Double T	40.5	7.9	46.1	147	2256	930	0.41	48.0	-	1.3
	CDT1-4-WA-A1			Girder	Sudden	Yes	Double T	40.5	7.9	46.1	147	2256	1640	0.73	48.0	487.5	2
	CDT1-4-WA-A2			Girder	Sudden	Yes	Double T	40.5	7.9	46.1	147	2256	1640	0.73	48.0	-	5.1
	CDT1-4-WA-A3			Girder	Sudden	Yes	Double T	40.5	7.9	46.1	147	2256	1240	0.55	48.0	-	2.5
	CDT1-4-WA-B1			Girder	Sudden	Yes	Double T	40.5	7.9	46.1	147	2256	1740	0.77	48.0	502.5	2.3
	CDT1-4-WA-B2			Girder	Sudden	Yes	Double T	40.5	7.9	46.1	147	2256	1350	0.60	48.0	-	3.3
	CDT1-4-WA-B3			Girder	Sudden	Yes	Double T	40.5	7.9	46.1	147	2256	1240	0.55	48.0	557.5	1.8
[19,20]	I-SCC30-1	CFRP Bar	Sanded	Beam	Gradual	Yes	150 × 250 × 3600	38.1	12.7	126.7	144	1765	613	0.32	30.4	-	1.478
	I-SCC30-2			Beam	Gradual	Yes	150 × 250 × 3601	38.1	12.7	126.7	144	1765	601	0.31	30.4	355.0	0.443
	I-SCC30-3			Beam	Gradual	Yes	150 × 250 × 3602	38.1	12.7	126.7	144	1765	726	0.37	41.0	-	0.784
	I-SCC30-4			Beam	Gradual	Yes	150 × 250 × 3603	38.1	12.7	126.7	144	1765	647	0.35	41.0	-	0.688
	II-SCC45-1			Beam	Gradual	Yes	150 × 250 × 3604	38.1	12.7	126.7	144	1765	807	0.43	35.0	-	1.393
	II-SCC45-2			Beam	Gradual	Yes	150 × 250 × 3605	38.1	12.7	126.7	144	1765	884	0.46	35.0	505.0	1.406
	II-SCC45-3			Beam	Gradual	Yes	150 × 250 × 3606	38.1	12.7	126.7	144	1765	841	0.45	35.0	530.0	1.432
	II-SCC45-4			Beam	Gradual	Yes	150 × 250 × 3607	38.1	12.7	126.7	144	1765	805	0.43	35.0	-	1.320
	III-SCC60-1			Beam	Gradual	Yes	150 × 250 × 3608	38.1	12.7	126.7	144	1765	980	0.54	30.4	665.0	2.164
	III-SCC60-2			Beam	Gradual	Yes	150 × 250 × 3609	38.1	12.7	126.7	144	1765	1097	0.58	30.4	-	2.572
	III-SCC60-3			Beam	Gradual	Yes	150 × 250 × 3610	38.1	12.7	126.7	144	1765	1008	0.54	41.0	695.0	2.105
	III-SCC60-4			Beam	Gradual	Yes	150 × 250 × 3611	38.1	12.7	126.7	144	1765	1052	0.57	41.0	680.0	2.508
	IV-N30-1			Beam	Gradual	Yes	150 × 250 × 3612	38.1	12.7	126.7	144	1765	635	0.33	37.0	300.0	0.539
	IV-N60-2			Beam	Gradual	Yes	150 × 250 × 3613	38.1	12.7	126.7	144	1765	1168	0.63	37.0	-	2.075
	IV-N60-3			Beam	Gradual	Yes	150 × 250 × 3614	38.1	12.7	126.7	144	1765	1121	0.60	37.0	580.0	1.826
	IV-N60-4			Beam	Gradual	Yes	150 × 250 × 3615	38.1	12.7	126.7	144	1765	1153	0.62	37.0	590.0	1.908

**Table A3:** Experimental parameters of specimens with pretensioned AFRP reinforcement.

Reference	Specimen No	FRP Type	FRP Surface	Specimen Type	Release Type	Shear Reinforcement	Specimen Dimensions (b × h × l, mm)	c, mm	Ø, mm	A_p_, mm^2^	E_p_, GPa	f_pu_, MPa	f_pi_, MPa	f_pi_/f_pu_	f_ci_, MPa	L_t_, mm	Slip, mm
[21]	7.5S-N-50-1	AFRP Arapee Tendon	Sanded	Prism	Gradual	No	50 × 50 × 1000	21.3	7.5	22.2	91	3000	1487	0.50	54.6	-	-
	7.5S-N-60-1		Sanded	Prism	Gradual	No	60 × 60 × 1000	26.3	7.5	22.2	91	3000	1487	0.50	54.6	135.0	0.625
	7.5S-N-70-1		Sanded	Prism	Gradual	No	70 × 70 × 1000	31.3	7.5	22.2	91	3000	1487	0.50	54.6	135.0	0.488
	7.5S-N-50-2		Sanded	Prism	Gradual	No	50 × 50 × 1000	21.3	7.5	22.2	91	3000	1487	0.50	54.6	220.0	1.239
	7.5S-N-60-2		Sanded	Prism	Gradual	No	60 × 60 × 1000	26.3	7.5	22.2	91	3000	1487	0.50	54.6	110.0	0.388
	7.5S-N-60-2		Sanded	Prism	Gradual	No	60 × 60 × 1000	26.3	7.5	22.2	91	3000	1487	0.50	54.6	185.0	1.15
	7.5S-N-70-2		Sanded	Prism	Gradual	No	70 × 70 × 1000	31.3	7.5	22.2	91	3000	1487	0.50	54.6	135.0	0.456
	7.5S-N-50-3		Sanded	Prism	Gradual	Fibers	50 × 50 × 1000	21.3	7.5	22.2	91	3000	1487	0.50	54.6	135.0	0.374
	7.5-S-N-50-4		Sanded	Prism	Gradual	Fibers	50 × 50 × 1000	21.3	7.5	22.2	91	3000	1487	0.50	54.6	160.0	0.681
	7.5S-N-50-5		Sanded	Prism	Gradual	Fibers	50×50×1000	21.3	7.5	22.2	91	3000	1487	0.50	54.6	160.0	0.67
	7.5S-H-45-1		Sanded	Prism	Gradual	No	45 × 45 × 1000	18.8	7.5	22.2	91	3000	1487	0.50	81.5	110.0	0.312
	7.5S-H-50-1		Sanded	Prism	Gradual	No	50 × 50 × 1000	21.3	7.5	22.2	91	3000	1487	0.50	81.5	135.0	0.398
	7.5S-H-50-2		Sanded	Prism	Gradual	No	50 × 50 × 1000	21.3	7.5	22.2	91	3000	1487	0.50	81.5	135.0	0.466
	5.3S-N-45-1		Sanded	Prism	Gradual	No	45 × 45 × 1000	19.9	5.3	11.1	91	3000	1487	0.50	54.6	85.0	0.378
	5.3E-N-45-1		Expancel	Prism	Gradual	No	45 × 45 × 1000	19.9	5.3	11.1	91	3000	1487	0.50	54.6	225.0	0.883
	5.3ES-N-45-1		Expancel Sanded	Prism	Gradual	No	45 × 45 × 1000	19.9	5.3	11.1	91	3000	1487	0.50	54.6	85.0	0.477
	5.3S-N-40-1		Sanded	Prism	Gradual	No	40 × 40 × 1000	17.4	5.3	11.1	91	3000	1487	0.50	54.6	85.0	0.27
	5.3E-N-40-1		Expancel	Prism	Gradual	No	40 × 40 × 1000	17.4	5.3	11.1	91	3000	1487	0.50	54.6	200.0	0.931
	5.3S-N-35-1		Sanded	Prism	Gradual	No	35 × 35 × 1000	14.9	5.3	11.1	91	3000	1487	0.50	54.6	85.0	0.376
	5.3S-N-30-1		Sanded	Prism	Gradual	No	30 × 30 × 1000	12.4	5.3	11.1	91	3000	1487	0.50	54.6	-	-
	5.3E-N-35-1		Expancel	Prism	Gradual	No	35 × 35 × 1000	14.9	5.3	11.1	91	3000	1487	0.50	54.6	185.0	0.811
	5.3E-N-30-1		Expancel	Prism	Gradual	No	30 × 30 × 1000	12.4	5.3	11.1	91	3000	1487	0.50	54.6	-	-
	5.3ES-N-35-1		Expancel Sanded	Prism	Gradual	No	35 × 35 × 1000	14.9	5.3	11.1	91	3000	1487	0.50	54.6	110.0	0.655
	5.3ES-N-30-1		Expancel Sanded	Prism	Gradual	No	30 × 30 × 1000	12.4	5.3	11.1	91	3000	1487	0.50	54.6	-	-
	5.3S-N-40-2		Sanded	Prism	Gradual	No	40 × 40 × 1000	17.4	5.3	11.1	91	3000	1487	0.50	54.6	-	-
	5.3S-N-35-2		Sanded	Prism	Gradual	No	35 × 35 × 1000	14.9	5.3	11.1	91	3000	1487	0.50	54.6	85.0	0.342
	5.3-ES-N-40-1		Expancel Sanded	Prism	Gradual	No	40 × 40 × 1000	17.4	5.3	11.1	91	3000	1487	0.50	54.6	135.0	0.633
	5.3ES-N-40-2		Expancel Sanded	Prism	Gradual	No	40 × 40 × 1000	17.4	5.3	11.1	91	3000	1487	0.50	54.6	-	-
	5.3ES-N-35-2		Expancel Sanded	Prism	Gradual	No	35 × 35 × 1000	14.9	5.3	11.1	91	3000	1487	0.50	54.6	110.0	0.568
	5.3ES-N-30-2		Expancel Sanded	Prism	Gradual	No	30 × 30 × 1000	12.4	5.3	11.1	91	3000	1487	0.50	54.6	-	-
[22]	FIB1/1a	FiBRA	Braided	Beam	Gradual	No	100 × 200 × 2800	58.2	3.7	11.0	69	1427	873	0.61	56.0	118	0.74
	FIB1/2a			Beam	Gradual	No	100 × 200 × 2800	58.2	3.7	11.0	69	1427	900	0.63	56.0	122	0.79
	FIB1/3a			Beam	Gradual	No	100 × 200 × 2800	58.2	3.7	11.0	69	1427	891	0.62	56.0	94	0.56
	FIB1/1b			Beam	Gradual	No	100 × 200 × 2800	58.2	3.7	11.0	69	1427	836	0.59	56.0	118.0	0.71
	FIB1/2b			Beam	Gradual	No	100 × 200 × 2800	58.2	3.7	11.0	69	1427	900	0.63	56.0	148.0	0.92
	FIB1/3b			Beam	Gradual	No	100 × 200 × 2800	58.2	3.7	11.0	69	1427	891	0.62	56.0	112.0	0.69
	FIB2/1a			Beam	Gradual	No	100 × 200 × 2800	58.2	3.7	11.0	69	1427	909	0.64	56.3	117	0.72
	FIB2/2a			Beam	Gradual	No	100 × 200 × 2800	58.2	3.7	11.0	69	1427	945	0.66	56.3	121	0.81
	FIB2/3a			Beam	Gradual	No	100 × 200 × 2800	58.2	3.7	11.0	69	1427	955	0.67	56.3	114	0.7
	FIB2/1b			Beam	Gradual	No	100 × 200 × 2800	58.2	3.7	11.0	69	1427	873	0.61	56.3	84.0	0.5
	FIB2/2b			Beam	Gradual	No	100 × 200 × 2800	58.2	3.7	11.0	69	1427	918	0.64	56.3	104.0	0.69
	FIB2/3b			Beam	Gradual	No	100×200×2800	58.2	3.7	11.0	69	1427	945	0.66	56.3	113.0	0.72
	FAB/1a			Beam	Gradual	No	100 × 200 × 2800	58.2	3.7	11.0	69	1427	918	0.64	58.0	107	0.65
	FAB/2a			Beam	Gradual	No	100 × 200 × 2800	58.2	3.7	11.0	69	1427	918	0.64	58.0	116	0.76
	FAB/3a			Beam	Gradual	No	100 × 200 × 2800	58.2	3.7	11.0	69	1427	900	0.63	58.0	82	0.53
	FAB/1b			Beam	Gradual	No	100 × 200 × 2800	58.2	3.7	11.0	69	1427	900	0.63	58.0	89.0	0.58
	FAB/2b			Beam	Gradual	No	100 × 200 × 2800	58.2	3.7	11.0	69	1427	945	0.66	58.0	117.0	0.78
	FAB/3b			Beam	Gradual	No	100 × 200 × 2800	58.2	3.7	11.0	69	1427	918	0.64	58.0	97.0	0.64
[22]	TIB1/1a	Technora	Spirally wounded	Beam	Gradual	No	100 × 200 × 2800	68.0	4.0	12.6	54	1802	1151	0.64	56.0	45	0.47
	TIB1/2a			Beam	Gradual	No	100 × 200 × 2800	68.0	4.0	12.6	54	1802	1167	0.65	56.0	94	0.96
	TIB1/1b			Beam	Gradual	No	100 × 200 × 2800	68.0	4.0	12.6	54	1802	1135	0.63	56.0	54.0	0.53
	TIB1/2b			Beam	Gradual	No	100 × 200 × 2800	68.0	4.0	12.6	54	1802	1190	0.66	56.0	78.0	0.8
	TIB2/1a			Beam	Gradual	No	100 × 200 × 2800	68.0	4.0	12.6	54	1802	1175	0.65	56.3	55	0.58
	TIB2/2a			Beam	Gradual	No	100 × 200 × 2800	68.0	4.0	12.6	54	1802	1151	0.64	56.3	59	0.6
	TIB2/1b			Beam	Gradual	No	100 × 200 × 2800	68.0	4.0	12.6	54	1802	1167	0.65	56.3	53.0	0.53
	TIB2/2b			Beam	Gradual	No	100 × 200 × 2800	68.0	4.0	12.6	54	1802	1151	0.64	56.3	58.0	0.6
	TAB/1a			Beam	Gradual	No	100 × 200 × 2800	68.0	4.0	12.6	54	1802	1151	0.64	58.0	59	0.57
	TAB/2a			Beam	Gradual	No	100 × 200 × 2800	68.0	4.0	12.6	54	1802	1159	0.64	58.0	61	0.6
	TAB/1b			Beam	Gradual	No	100 × 200 × 2800	68.0	4.0	12.6	54	1802	1151	0.64	58.0	55.0	0.58
	TAB/2b			Beam	Gradual	No	100 × 200 × 2800	68.0	4.0	12.6	54	1802	1159	0.64	58.0	47.0	0.5

**Table A4:** Experimental parameters of specimens with pretensioned BFRP reinforcement.

Reference	Specimen No	FRP Type	FRP Surface	Specimen Type	Release Type	Shear Reinforcement	Specimen Dimensions (b × h × l, mm)	c, mm	Ø, mm	A_p_, mm^2^	E_p_, GPa	f_pu_, MPa	f_pi_, MPa	f_pi_/f_pu_	f_ci_, MPa	L_t_, mm	Slip, mm
[23]	B1S	BFRP Bar	Sanded	Beam	Sudden	No	3000 × 100 × 200	46	8	50.24	44	1126	393	0.34	27	350	1.83
	B1N			Beam	Sudden	No	3000 × 100 × 201	46	8	50.24	44	1126	384	0.32	27	500	1.91
	B2S			Beam	Sudden	No	3000 × 100 × 202	46	8	50.24	44	1126	369	0.31	27	250	1.99
	B2N			Beam	Sudden	No	3000 × 100 × 203	46	8	50.24	44	1126	375	0.31	27	400	2.79

## Appendix B

**Table A5:** The influence of α on the statistical results of L_t.teor_/L_t.exp_ of CFCC strands.

α	3	3.1	3.2	3.3	3.4	3.45	3.5	3.6	3.7	3.8	3.9	4
M	0.909	0.939	0.969	1.000	1.030	1.046	1.060	1.091	1.121	1.151	1.182	1.212
STD	0.189	0.195	0.201	0.208	0.214	0.217	0.220	0.226	0.233	0.239	0.245	0.252
COV, %	20.8	20.8	20.8	20.8	20.8	20.8	20.8	20.8	20.8	20.8	20.8	20.8
N_total_	27	27	27	27	27	27	27	27	27	27	27	27
OV	6	9	12	13	15	15	15	18	20	21	22	22
UV	21	18	15	14	12	12	12	9	7	6	5	5

**Table A6:** The influence of α on the statistical results of L_t.teor_/L_t.exp_ of CFRP bars.

α	2	2.1	2.2	2.3	2.4	2.46	2.5	2.54	2.6	2.7	2.8	2.9	3
M	0.813	0.854	0.894	0.935	0.976	1.000	1.016	1.034	1.057	1.098	1.138	1.179	1.220
STD	0.143	0.150	0.157	0.164	0.171	0.18	0.18	0.182	0.186	0.193	0.200	0.207	0.214
COV, %	17.6	17.6	17.6	17.6	17.6	17.6	17.6	17.6	17.6	17.6	17.6	17.6	17.6
N_total_	27	27	27	27	27	27	27	27	27	27	27	27	27
OV	2	6	10	11	12	13	15	16	16	19	21	22	23
UV	26	21	17	16	15	14	12	11	11	8	6	5	4

**Table A7:** The influence of α on the statistical results of L_t.teor_/L_t.exp_ of AFRP Arapree bars.

α	3.5	3.6	3.7	3.8	3.84	3.9	4	4.043	4.1	4.2	4.3	4.4	4.5
M	0.912	0.938	0.964	0.990	1.001	1.016	1.042	1.053	1.068	1.094	1.120	1.147	1.173
STD	0.212	0.218	0.224	0.230	0.232	0.236	0.242	0.245	0.248	0.254	0.260	0.266	0.272
COV, %	23.2	23.2	23.2	23.2	23.2	23.2	23.2	23.2	23.2	23.2	23.2	23.2	23.2
N_total_	23	23	23	23	23	23	23	23	23	23	23	23	23
OV	6	8	10	11	12	12	13	13	14	15	15	15	15
UV	17	15	13	12	11	11	10	10	9	8	8	8	8

**Table A8:** The influence of α on the statistical results of L_t.teor_/L_t.exp_ of AFRP Fibra and Technora bars.

α	2.040	2.050	2.060	2.070	2.080	2.090	2.095	2.097	2.1	2.110	2.120	2.130	2.140
M	0.974	0.979	0.983	0.988	0.993	0.998	1.000	1.001	1.002	1.007	1.012	1.017	1.022
STD	0.032	0.032	0.033	0.033	0.033	0.033	0.033	0.033	0.033	0.033	0.034	0.034	0.034
COV, %	3.3	3.3	3.3	3.3	3.3	3.3	3.3	3.3	3.3	3.3	3.3	3.3	3.3
N_total_	30	30	30	30	30	30	30	30	30	30	30	30	30
OV	9	11	12	15	15	15	15	16	16	17	19	19	20
UV	21	19	18	15	15	15	15	14	14	13	11	11	10

**Table A9:** The influence of α on the statistical results of L_t.teor_/L_t.exp_ of all AFRP bars.

α	2	2.1	2.2	2.3	2.4	2.5	2.6	2.61	2.7	2.8	2.9	2.941	3
M	0.767	0.805	0.843	0.882	0.920	0.958	0.997	1.000	1.035	1.073	1.112	1.127	1.150
STD	0.232	0.244	0.255	0.267	0.278	0.290	0.302	0.303	0.313	0.325	0.336	0.341	0.348
COV, %	30.3	30.3	30.3	30.3	30.3	30.3	30.3	30.3	30.3	30.3	30.3	30.3	30.3
N_total_	53	53	53	53	53	53	53	53	53	53	53	53	53
OV	0	16	27	30	30	30	30	30	31	32	32	34	34
UV	53	37	26	23	23	23	23	23	22	21	21	19	19

**Table A10:** The influence of α on the statistical results of L_t.teor_/L_t.exp_ of BFRP bars.

α	1	1.1	1.2	1.3	1.4	1.43	1.5	1.57	1.6	1.7	1.8	1.9	2
M	0.698	0.767	0.837	0.907	0.977	0.998	1.046	1.093	1.116	1.186	1.256	1.326	1.395
STD	0.229	0.252	0.275	0.298	0.321	0.328	0.344	0.360	0.367	0.390	0.413	0.436	0.459
COV, %	32.9	32.9	32.9	32.9	32.9	32.9	32.9	32.9	32.9	32.9	32.9	32.9	32.9
N_total_	4	4	4	4	4	4	4	4	4	4	4	4	4
OV	0	1	1	2	2	2	2	2	2	2	3	3	3
UV	4	3	3	2	2	2	2	2	2	2	1	1	1

M—mean L_t.teor_/L_t.exp_, STD—standard deviation, COV—coefficient of variation, N_total_—the total number of specimens, OV—overestimated values, UV—underestimated values.
